# Development of the COVID-19 Real-Time Information System for Preparedness and Epidemic Response (CRISPER), Australia

**DOI:** 10.3389/fpubh.2021.753493

**Published:** 2021-11-11

**Authors:** Emma Field, Amalie Dyda, Michael Hewett, Haotian Weng, Jingjing Shi, Stephanie Curtis, Charlee Law, Lisa McHugh, Meru Sheel, Jess Moore, Luis Furuya-Kanamori, Priyanka Pillai, Paul Konings, Michael Purcell, Nigel Stocks, Graham Williams, Colleen L. Lau

**Affiliations:** ^1^National Centre for Epidemiology and Population Health, Research School of Population Health, ANU College of Health and Medicine, Australian National University, Canberra, ACT, Australia; ^2^Global and Tropical Health Division, Menzies School of Health Research, Charles Darwin University, Darwin, NT, Australia; ^3^School of Public Health, University of Queensland, Herston, QLD, Australia; ^4^The National Centre for Geographic Resources & Analysis in Primary Health Care (GRAPHC), Research School of Population Health, Australian National University, Canberra, ACT, Australia; ^5^Software Innovation Institute, Australian National University, Canberra, ACT, Australia; ^6^School of Computing, Australian National University, Canberra, ACT, Australia; ^7^UQ Centre for Clinical Research, University of Queensland, Herston, QLD, Australia; ^8^Doherty Institute, Melbourne, VIC, Australia; ^9^Discipline of General Practice, University of Adelaide, Adelaide, SA, Australia; ^10^Australian Partnership for Preparedness Research on InfectiouS Disease Emergencies (APPRISE), The Peter Doherty Institute for Infection and Immunity, Melbourne, VIC, Australia; ^11^Research School of Population Health, ANU College of Health and Medicine, Australian National University, Canberra, ACT, Australia

**Keywords:** infectious disease, information sources, epidemics, information management, data visualization

## Abstract

Accurate and current information has been highlighted across the globe as a critical requirement for the COVID-19 pandemic response. To address this need, many interactive dashboards providing a range of different information about COVID-19 have been developed. A similar tool in Australia containing current information about COVID-19 could assist general practitioners and public health responders in their pandemic response efforts. The COVID-19 Real-time Information System for Preparedness and Epidemic Response (CRISPER) has been developed to provide accurate and spatially explicit real-time information for COVID-19 cases, deaths, testing and contact tracing locations in Australia. Developed based on feedback from key users and stakeholders, the system comprises three main components: (1) a data engine; (2) data visualization and interactive mapping tools; and (3) an automated alert system. This system provides integrated data from multiple sources in one platform which optimizes information sharing with public health responders, primary health care practitioners and the general public.

## Introduction

The rapid global spread of COVID-19 has underscored the importance of rapid information sharing of accurate data to assist with outbreak response and public health decision making. To address this ongoing need for accurate real-time data (1), numerous COVID-19 dashboards have been created worldwide for many different purposes including tracking cases and deaths by place and time (2–4), mapping telehealth services (5), monitoring areas of vulnerability using information such as infection rates and population attributes (6), tracking information on COVID-19 clinical trials (7), genomic surveillance (8) and vaccine rollout (9).

In Australia, the federal and jurisdictional Departments of Health provide daily updates on COVID-19 through mostly static graphs and tables (10), and information is available from other sources such as the National Notifiable Disease Surveillance System (NNDSS) and the COVIDSafe smartphone application (11, 12). Due to the federated nature of Australia's health system, each state and territory provides data to the public on COVID-19 cases, deaths, testing and contact tracing locations on their health department websites (9, 13). These data are however, in different formats, and vary in their level of granularity, which creates a barrier for sharing and comparing data between the states and territories.

Most publicly available resources on Australia's health department websites also have limited capability to enable information seekers to interrogate the data and ask epidemiologically sound questions related to time, place and person. While some media websites and other organizations outside of government have developed dashboards, they have scraped data from health department websites to generate their own databases, with reported data often aggregated at state level and not of sufficient detail to inform action (14).

Currently, there are major barriers in accessing detailed real-time data on COVID-19 from official sources in Australia. There is currently no national, real-time, public-facing dashboard available in a format that allows users to interrogate the data and ask specific epidemiological questions. Such information would be useful for various types of users. General practitioners (GPs) and other Allied Health Professionals (AHP) could use real-time data for risk assessment for patients and staff, help decide when to shift to telehealth consultations during high-risk times, determine if patients have been to a contact tracing alert location, and alert vulnerable patients if there is a high risk of local transmission. More detailed data could also assist public health responders with swift visual situational awareness, contact tracing, provide data visualization (of cases, clusters, and contact tracing locations), generate epidemiological reports, and help to identify areas with low testing and/or vaccination rates, both within and outside their jurisdiction. In addition, up to date contact tracing alert information shown spatially could better assist the public to understand whether they have attended an exposure site, thereby enabling them to more promptly present for testing and/or avoid visiting areas with high risk of transmission.

To meet the information needs of public health responders, primary health care workers and the public, we developed the COVID-19 Real-time Information System for Preparedness and Epidemic Response (CRISPER) (15). In this paper, we describe the development of CRISPER, which includes a suite of interactive online dashboards, interactive mapping tools, and an automated alert system for COVID-19 in Australia.

## Methods

### Development of Crisper

#### Stakeholder Engagement

During the design of CRISPER, we consulted with a wide range of stakeholders such as public health physicians and epidemiologists in state and territory health departments, Primary Health Care Networks, GPs, telemedicine doctors, and researchers. We also engaged with the National Aboriginal Community Health Organization and the Queensland Aboriginal and Islander Health Council to understand the needs of First Nations health organizations. These consultations focused on the information needs of diverse potential users, how they currently access information, challenges with accessing timely information, the information gaps, and the desired functionalities from a national information system such as CRISPER. The project proposal was sent to Communicable Diseases Network Australia (CDNA), a committee in Australia that provides national public health co-ordination and leadership and support best practice for the prevention and control of communicable diseases. The feedback from CDNA helped the project team understand to the usability of a platform like CRISPER and understand the information gathering needs that CRISPER can fulfill.

#### System Architecture

**Figure 1:**
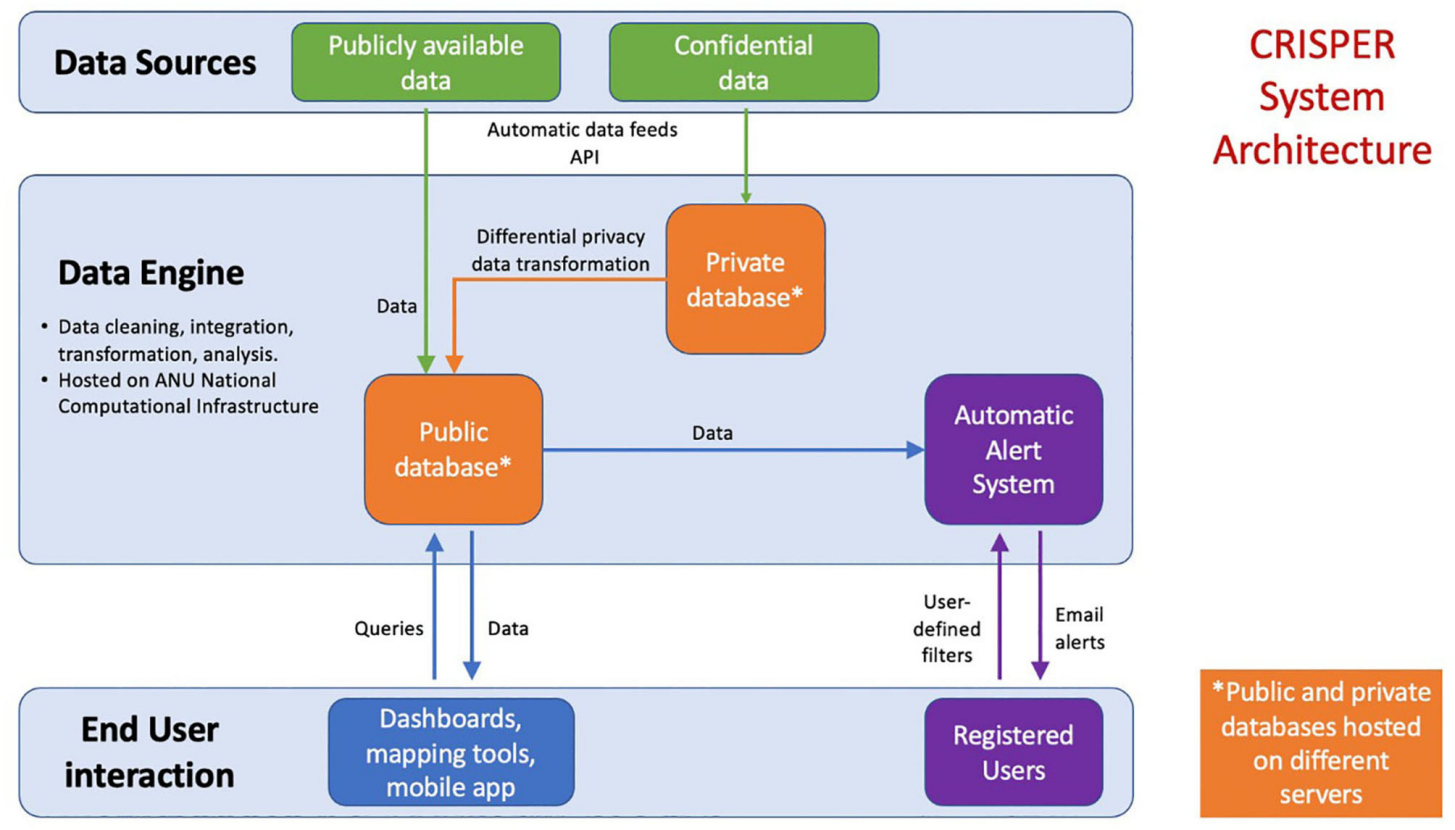
System architecture of the CRISPER system.

We engaged an independent consulting company to undertake a systems analysis to inform the design of CRISPER. Representatives from the independent consulting company conducted semi-structured interviews with potential users (*n* = 29) including primary health care, data specialists and public health professionals to determine user needs and an appropriate system architecture. Participants were sent a link to a short online survey asking about the usefulness of the potential features of CRISPER (e.g., mapping tool, automated alerts system). The results from the interviews and survey were synthesized and summarized by the independent consulting company. Results from the systems analysis recommended that CRISPER should consist of three main components: (1) a data engine where data from various sources are assembled, cleaned, summarized, and where appropriate confidentiality measures are put in place; (2) data visualization and interactive mapping tools; and (3) an automated alert system (Figure 1).

#### System Novelty

The CRISPER website (https://crisper.net.au) provides access to all publicly available CRISPER project content, including dashboards, charts, mapping tools, background information, instructional videos, and contains a number of novel and beneficial features (Box 1).

Box 1 **Features of the CRISPER system.**

Provides national, (near) real-time, public facing data in relation to COVID-19Interactive mapping of cases and contact tracing alert locations, including a mobile-enabled web app

An interface which allows users to interrogate data and ask specific questionsThe use of differential privacy to protect confidentiality of individual-level data

Automated alerts of cases, tests and contact tracing locations, customized to the user's geographical location of interest

#### Data Engine and Data Sharing

The data engine is the integration point for the sources of health and geospatial data. It combines data from multiple and often vastly different public sources into a common format (normalization). The data engine is hosted by the National Computational Infrastructure (NCI) at the Australian National University (ANU).

Examples of the types of data collected include daily COVID-19 cases by jurisdiction, daily COVID-19 testing numbers by postcode and contact tracing locations.

A suite of carefully tuned data scrapers have been implemented to parse the data formats of the many different public sources. The data from multiple sources are then reshaped into a normalized format. This entails rearranging data from states and territories and considering differences in the type, format and amount of data that are available. The State and Territory datasets are then combined into harmonized Australia-wide datasets and used for the applications utilizing the data engine.

Collecting data from multiple public sources in this way presents challenges in terms of the lag between the data becoming available in the public sites and being collected into the data engine, and the lag between governments announcing updates and those updates becoming available on their websites. Due to staffing and resourcing shortages, data on health department websites are often not updated over weekends and holidays, and the lag can sometimes be multiple days. The data engine monitors all source data sites hourly to ensure it is as recent as feasible. All updates are logged using time stamps enabling the data engine to report on the timeliness of the data.

Data sharing and transparency of public health agencies is crucial in ensuring the integrity of the data for those who are utilizing CRISPER. The data are only as up-to-date and as accurate as the agencies have made available through their data sharing. The accuracy of the data on the dashboard is paramount due to the rapid and large-scale public health response measures implemented in Australia when there are any variations in the relatively low case numbers. The dashboard aims to optimize information sharing and interpretation to augment the knowledge gain for end users.

All collected and value-added data can be accessed through the CRISPER home page at https://crisper.net.au. The data schemas, meta-data, and the actual data are available through a standard JSON-based API (Application Programming Interface), allowing the source and value-added data to be openly reviewed and programmatically utilized. We have utilized this data with the CRISPER suite of technologies and these data are regarded as the “single source of truth” for the suite. The data engine is available to independent developers, including government.

#### Data Sources

The distributed network of public health data systems across jurisdictions makes it challenging to access line-listed data from each and every system for secondary purposes. To date, the CRISPER suite of technologies has been developed using publicly available data. The CRISPER platform has the potential to be expanded if more granular data (e.g., line-list data) can be made available.

Data are not currently available for all states and territories or at all resolutions (Table 1). Consequently, different layers and charts include information for different areas at different resolutions. Data in free text reports, in graphical format only, or protected by business intelligence and analytics systems such as Tableau or Power BI are generally unusable as a data source for the dashboards.

**Table 1:** Current data sources (as of June 2021) for the COVID-19 Real-time Information System for Preparedness and Epidemic Response (CRISPER).

States and territories^*^	**Data type**	**Source of data**	**Spatial resolution of cases (daily)**	**Spatial resolution of tests (daily)**	**Spatial resolution of deaths (daily)**	**Alert Locations**
NSW	Cases, testing, contact tracing alert locations	.csv and .json sources available on the NSW government website (16)	Postcode, local government area (LGA), Primary Health Network	Postcode and LGA	Not available^a^	Reported by address, latitude/longitude
VIC	Cases and contact tracing alert locations	.csv sources available on the VIC government websites for cases and contact tracing locations (17, 18)	Postcode, LGA	Not available^a^	Not available^a^	Reported by address and postcode
WA	Cases, deaths and contact tracing alert locations	.json sources available on the WA government websites for cases and contact tracing locations (19)	State	Not available^a^	State	Reported by address
ACT, NT, SA, TAS, QLD	Cases, deaths, testing, and contact tracing alert locations	Data published by https://www.covid19data.com.au/ under a Creative Commons 4 license (20). The raw data can be found on their GITHUB repository: https://raw.githubusercontent.com/M3IT/COVID-19_Data/master/Data/COVID_AU_state.csv ACT contact tracing alert locations are available on the ACT government website (21) NT contact tracing alert locations are available on the NT government website (22) QLD contact tracing alert locations are available on the QLD government website (23) SA contact tracing alert locations are available on the SA government website (24) TAS contact tracing alert locations are available on the TAS government website (25)	State	State	State	Reported by address
All states and territories	Regional boundaries for postcode areas and LGA Commonwealth declared LGA hotspots	Public datasets published by the Australian Bureau of Statistics (26) Commonwealth declared LGA hotspots are available on the Australian Government website (27)				

a *Not available—the specific data values were not available in the health department dataset. In these cases, the data for the state are accessed from the covid19data.com.au source*.

* *New South Wales (NSW), Victoria (Vic), Western Australia (WA), Australian Capital Territory (ACT), Northern Territory (NT), South Australia (SA), Tasmania (Tas), Queensland (QLD)*.

*Note: This table provides a current snapshot and is subject to change over time as data sources change. Please see https://crisper.net.au for the latest updates*.

Some jurisdictions provide postcode level data, others aggregated to local government areas (designated parts of a State or Territory over which incorporated local governing bodies have responsibility), and others to hospital districts. For contact tracing locations, for example, the New South Wales data includes longitude and latitude, whilst other jurisdictions do not provide such accurate locations, relying on shop names and street addresses. Where possible, the data engine utilizes a geo-coding capability to determine a longitude/latitude coordinate, as best as possible, given the street address.

In utilizing the data engine, it is important to recognize the jurisdictional differences and gaps in the data, as well as the data cleansing being undertaken by the data engine. Data cleansing involves removing special characters in the text, handling missing values, unifying date formats, and formatting the data as per specific requirements.

#### Data Privacy Provisions

A key issue when releasing health information publicly is maintaining the privacy and confidentiality of individuals. CRISPER currently uses only publicly available data. However, a request for line-listed case data from NNDSS is in progress. While CRISPER does not intend to use any identifying variables such as names, date of birth, or identification numbers, the NNDSS line-listed data could potentially be combined with other publicly available information to determine other demographics about that individual. For example, the media may report a case in a certain geographical area. Without sufficient privacy provisions, a person using an interactive dataset like CRISPER may be able to identify other characteristics about this case, such as age group or sex.

Differential privacy is a novel method for preserving privacy whereby small amounts of variation are added to the data in the data engine (28). The data presented will be matched to publicly available data where precision of case numbers is required. Other data that are not required to be so precise (e.g., test rates, proportions of cases by sex) will have a small but negligible variation presented so that no additional characteristics can be identified from a case. To test the use of differential privacy, a ‘dummy’ line-listed dataset was simulated using aggregated publicly available parameters from Queensland Health (29) as a proof of concept.

## Results

### Data Visualizations—Dashboards and Interactive Mapping Tools

The ESRI ArcGIS Online (30) platform was used to build the visualization and mapping tools for CRISPER due to its mapping capabilities. This online component allowed the creation of integrated websites and applications in a cloud-based environment which did not require the establishment or maintenance of a physical server. The system is scalable, and able to support several thousand users per minute.

The data visualizations are able to run on any common web-browser enabled device. The current version was designed for desktop users given that the initial target users of the system were envisioned to be office based, and interactive engagements for these detailed dashboards were unlikely to be user friendly in very small screens. Users on mobile devices may therefore find some components difficult to use due to the screen layout.

The visualization and mapping tools are presented as a series of online dashboards which are publicly available. The dashboards contain a combination of maps, charts, filters, lists, and other widgets that can interact to allow visualization and interaction with location-based data. Each CRISPER dashboard and interactive mapping tool is described below.

The dashboards source the health and geospatial data from data engine through the JSON-based API. A set of Python scripts download data via the data engine API and normalize the data into the corresponding format for each dashboard. The normalized data are then uploaded to the ESRI ArcGIS Online platform to update the dashboards. These Python scripts and data engine reside in the same cloud servers at the National Computational Infrastructure. The data update process is performed every hour.

#### National Summaries Dashboard

**Figure 2:**
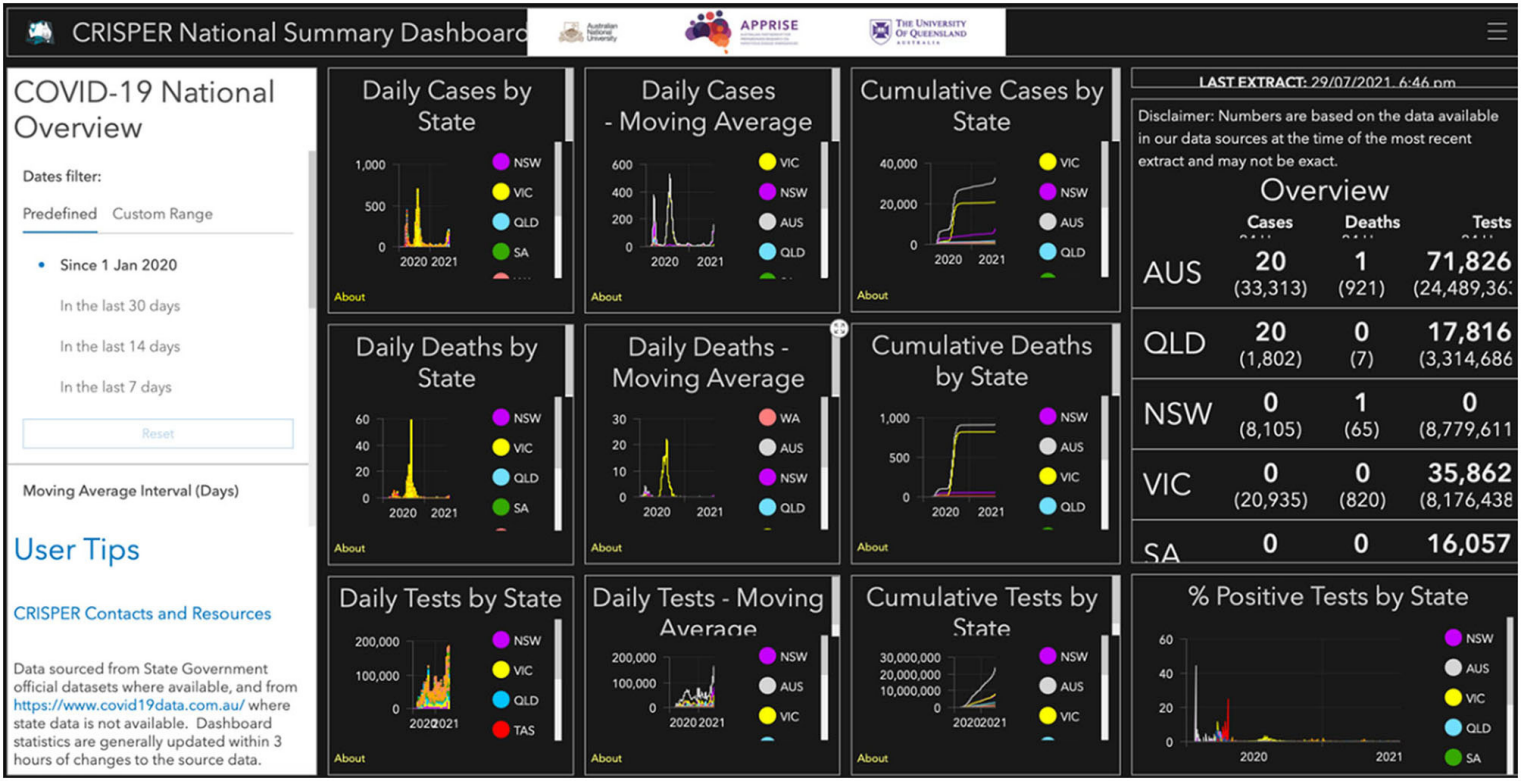
CRISPER National Summaries dashboard.

The National Summaries Dashboard displays an overview window with number of cases, deaths and tests in the last 24 h and days since last case by state/territory (Figure 2).

There are individual graphs for new daily cases, deaths and tests by count, moving average and cumulative total (cumulative cases are calculated by adding the daily case numbers for the specified period). The proportion of tests positive for COVID-19 are displayed (number of cases on a specific date/number of tests on date × 100). These figures can by filtered by date and state and the moving average for 7 days (sum of cases in the immediate 7 day period/7) or 14 days (sum of cases in the immediate 14 day period/14).

#### New South Wales Public Health Unit Dashboard

**Figure 3:**
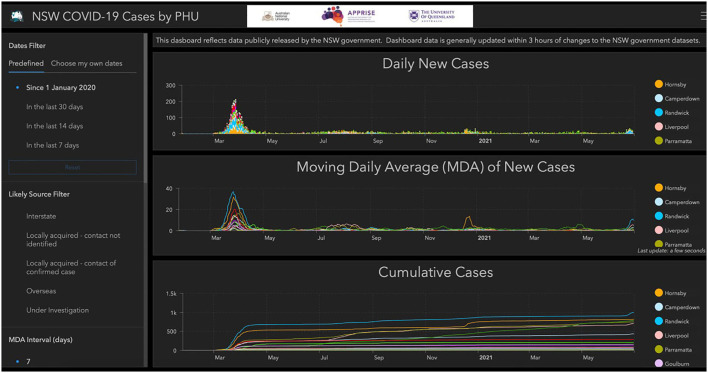
CRISPER dashboard for New South Wales Public Health Units.

The Public Health Unit (PHU) Dashboard presents graphs of new daily cases, a moving average of new cases and cumulative cases for each of the PHUs in New South Wales. These graphs can by filtered by date, likely source of infection and PHU, and the moving average is provided for 7 or 14 days (Figure 3).

#### Interactive Mapping Tool

**Figure 4:**
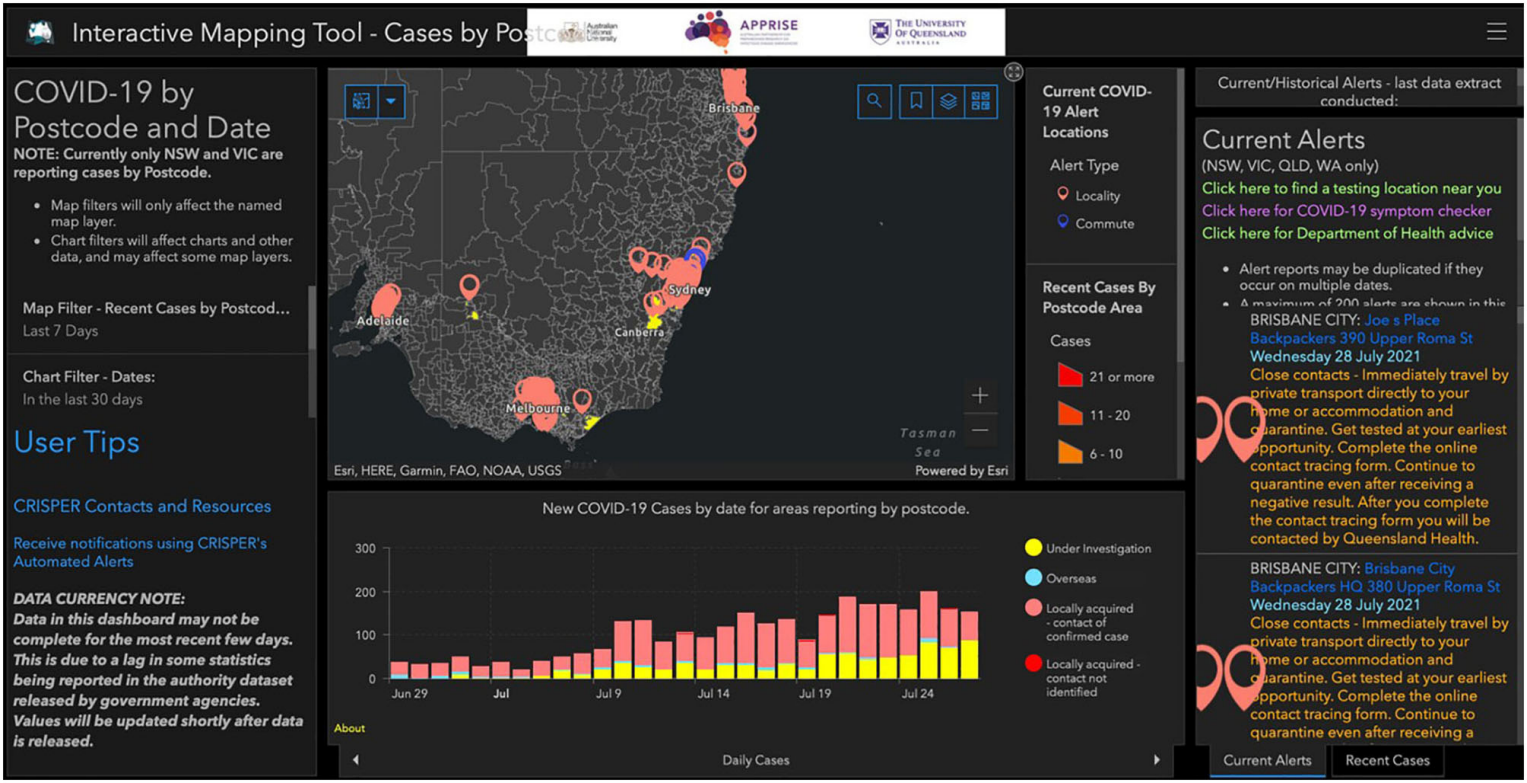
CRISPER interactive mapping tool.

This tool contains an interactive map of COVID-19 cases, with several additional windows that are linked to the data shown in the map: a graph of COVID-19 cases by day, cumulative cases, daily tests, a 7 day moving average for tests and cumulative tests; details of contact tracing alerts; and a graphical display of the number of cases and tests present in the map view area as a proportion of all reported cases and tests (Figure 4).

#### Contact Tracing Alerts Dashboard and Mobile-Enabled Web App

**Figure 5:**
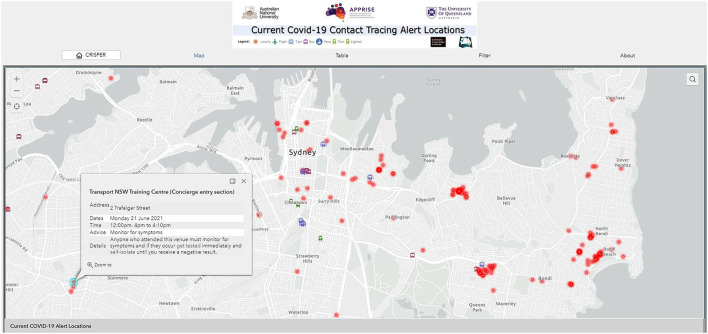
CRISPER contact tracing alerts dashboard and mobile-enabled web app.

This dashboard provides a map of all current contact tracing alert locations (i.e., locations where potential exposure to infectious COVID-19 cases may have occurred). The dashboard displays a map of Australia indicating each alert location (red points) and alerts on transport routes (icons for trains, buses, flights, ferries, etc.). The LGA hotspots, as declared by the Commonwealth government, are overlayed in the map. Clicking on the points and icons opens a pop-up menu with information on the venue name, address, date and time of exposure, and advice from the health department (e.g., get tested immediately) (Figure 5). This dashboard was developed with mobile phone capability (mobile.crisper.net.au) so users can easily check for new exposure locations and visualize areas with multiple alerts. Alerts can be filtered by date, state, and postcode. Search functions by postcode, suburb or address also allow alert locations to be easily identified.

### Automated Alerts System

Registered CRISPER users can set up automated alerts via https://crisper.net.au/. Different types of automated alerts are supported, including COVID-19 cases (e.g., have there been any new COVID-19 cases reported in my postcodes of interest in the past 14 days?), COVID-19 tests, and COVID-19 contact tracing locations (e.g., what are the current contact tracing locations in my postcodes of interest?).

**Figure 6:**
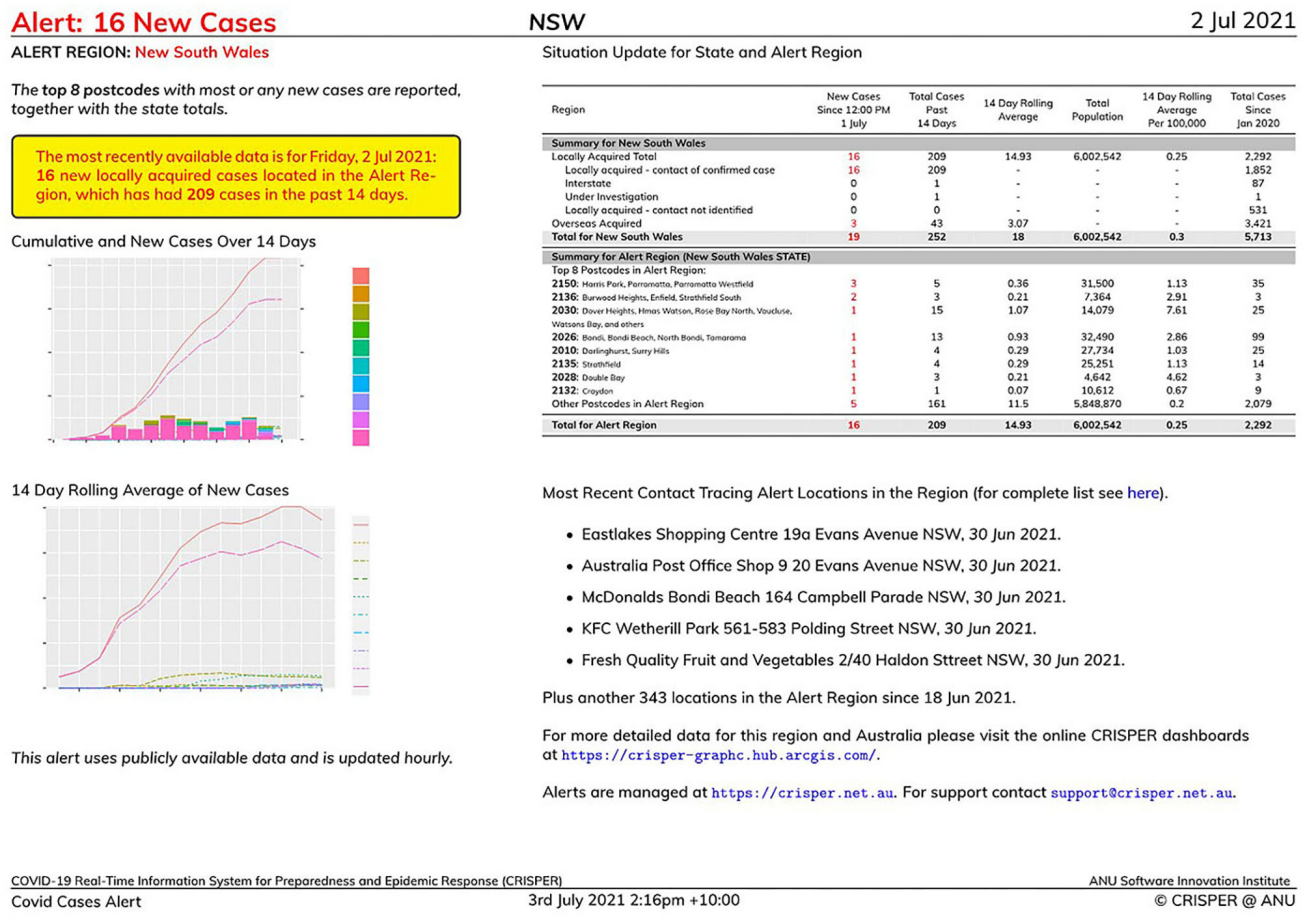
Example of a CRISPER automated alert report for the whole state of New South Wales.

Automated alerts for COVID-19 cases, tests or exposure sites can be created for various geographical regions of interest (state, post code/suburb, for all states and territories; in addition, LGA is available for Victoria and New South Wales; and Local Health District (LHD) is available for New South Wales). Many users have found the state level alerts to be of interest. An example of an alert for the whole of New South Wales is shown in Figure 6. The top eight postcodes according to the number of new reported COVID-19 cases are noted. The yellow highlighted box (which would be gray if there were no cases to report) notes 16 newly reported cases across the state and 209 cases over the past 14 days. The table on the right identifies that there were also three overseas acquired cases in addition to the 16 locally acquired cases. The two charts indicate the emerging emergency for the state (in this example it is for Sydney, noting the postcodes where the cases are being reported). The first chart covers the cumulative cases (line chart) and new cases (bar chart) for the last 14 days. The second chart reports the 14 day rolling average of new cases. A selection of the latest contact tracing locations is included in the bottom right region of the report, with a link to the contact tracing alerts dashboard or web app for the complete list of locations.

In addition to identifying a state as a region of interest the user may specify other geographic areas depending on their information needs, including postcodes, suburbs, PHUs, LGAs, and Local Health Districts. For example, a GP clinic may be interested in the postcodes around the clinic where their patients are likely to live, or a general member of the public may be interested in areas where they live and work.

Having identified the alert type and alert region, users of the Automated Alerts can then select the frequency of the alerts: daily (including weekends), weekday only, or once weekly. The alerts are then built as appropriate and emailed to the registered users' email address. Users of the Automated Alerts do not need to register in order to obtain an alert. The Demo option available (https://crisper.net.au/alerts_explorer) provides an immediate alert report using the same GUI interface. A specific date can be supplied to obtain a report of the situation on that date.

The underlying technology utilizes open-source software for the end-to-end processing. A combination of Python, R, and LaTeX are utilized to, respectively, obtain the required data from the CRISPER Data Engine, to statistically analyse that data, and to format the analysis.

### Usage Statistics and Media Coverage

The CRISPER dashboards were launched in October 2020. In the period 1st October to 30th June 2021, there were 10,849 online visits to the site to access the dashboards and maps.

**Figure 7:**
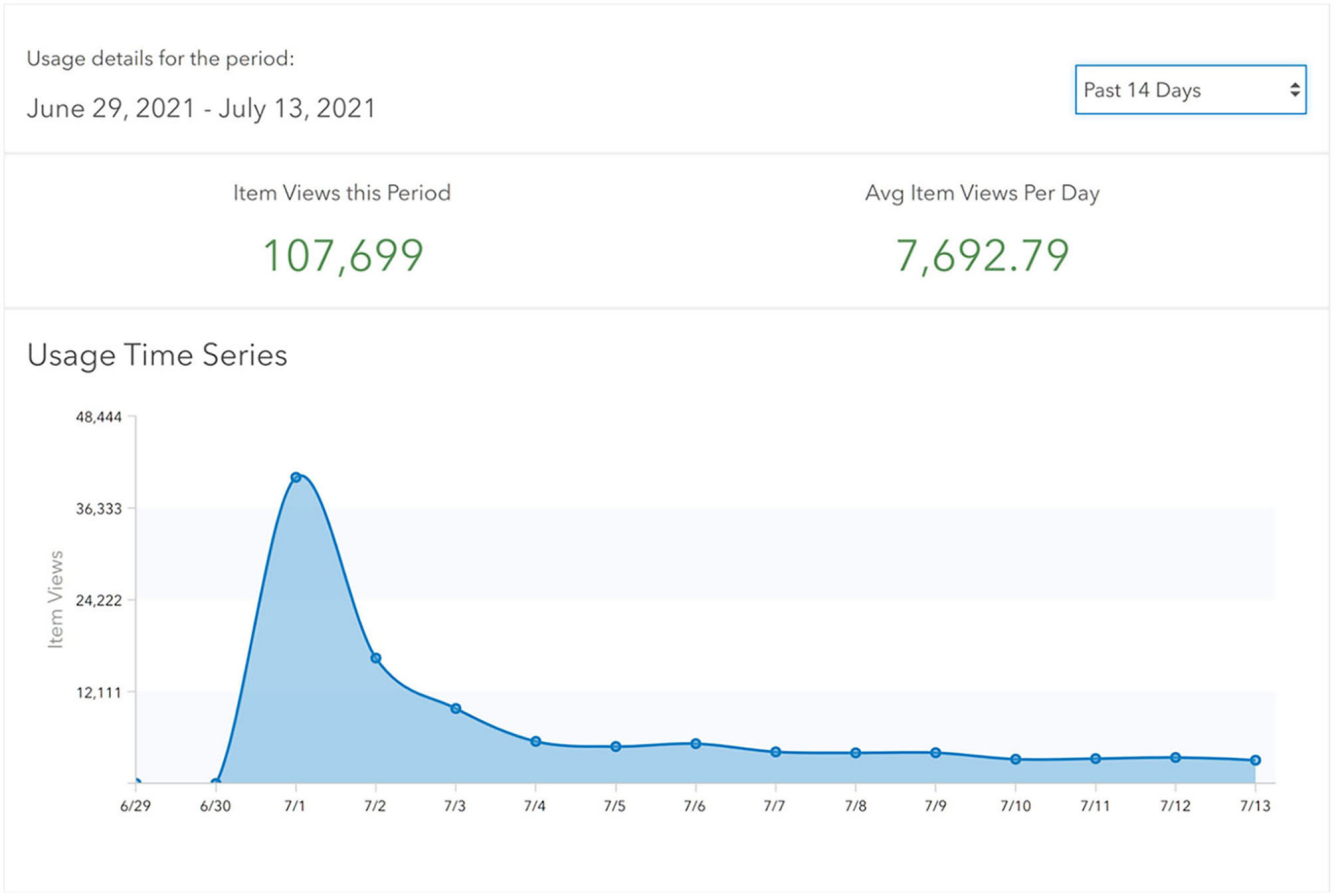
Usage Statistics for the Current COVID-19 Contact Tracing Alert Locations Web App for period: 1st July 2021 to 13th July 2021.

Attention on this tool has grown with the recent COVID-19 outbreaks in Australia, with CRISPER being featured in eight radio programs, six newspaper print articles, 157 online news articles and 46 television programs between the 1st and 5th July 2021. In the 5 days since the July 2021 launch and media release, there have been 107,699 visits to the CRISPER Contact Tracing Alert Locations Map Interface alone. Excluding the 2 days of high media attention (where visits exceeded 10,000 per hour for a 2 h period on the first day), access is now averaging over 500 per hour (between 08:00 and 20:00 h) (Figure 7).

## Discussion

Rapid sharing of accurate data is a critical part of any pandemic response. This was identified in Australia following the H1N1 2009 influenza pandemic (31), and was further highlighted in the Australian Health Sector Emergency Response Plan for Novel Coronavirus (32). Information in the current pandemic has been produced quickly and made available on an unprecedented scale through the internet and social media, using innovative technologies to optimize data sharing, visualization, and interpretation (33). Never before have public health responders and primary health care workers had such large amounts of information on the evolving epidemiology of a pandemic. However, there is a need for more coordinated and streamlined access to national data from official sources in Australia.

In Australia, a number of dashboards were created to address some information gaps and synthesize information at a national scale (34, 35). Although these dashboards are useful, they do not allow users to interrogate the data by time *and* place *and* source of infection nor do they present data in high spatial resolution, such as at the postcode level. Further, exposure site information is typically presented in tabular format on state/territory health websites, requiring users to scroll through long lists of exposure sites. CRISPER presents exposure sites spatially on an interactive map so users can easily identify exposure sites relevant to their location. Other visual dashboards suppress records in their maps to prevent individuals from being identified (36), however, CRISPER has been designed with differential privacy mechanisms that can be implemented to protect privacy when individual-level data are included.

At the national level, CRISPER creates an opportunity for useful real-time data visualization of COVID-19. Internationally, dashboards such as the Johns Hopkins COVID-19 dashboard (37) have had great utility in collecting, synthesizing and presenting a broad range of information to provide an indicator to researchers, health professionals and the public alike about global trends of the COVID-19 pandemic (38). The CRISPER data engine has similar capability to collect and process information from numerous sources by streamlining the key base variables that are reported across Australian states and territories. CRISPER provides information in much higher resolution than available through the Johns Hopkins COVID-19 dashboard (including details on local exposure sites and public health directions), plus the ability to interrogate data by time and place and source of infection. CRISPER is unique in that it provides the opportunity to spatially observe and report on national data from numerous official sources to describe the epidemiology of the pandemic and to analyse areas of concern and trends, with the ability to tailor this information to suit the user's local requirements. CRISPER also includes other useful spatial layers (e.g., locations of aged care facilities) that can be overlaid with data on cases, deaths, and testing.

At the state and territory level, CRISPER provides a reliable data source to visualize data across jurisdictions and may be of particular benefit to towns on state borders. Currently, acquiring comprehensive multi-jurisdictional data often relies on accessing different government websites, with some updated at different times during the day. Being able to view these data in real-time, particularly contact tracing alert locations, could be critical for swift cross-border situational awareness. This may be of benefit to individuals or organizations that have activities in multiple jurisdictions, such as managing human resources and supply chains across borders. Better visual displays are useful to help understand and communicate risk, and thereby improve compliance with public health directions.

At a local level, CRISPER can provide an immediate snapshot to identify clusters and potential areas of concern that require additional public health resources to minimize the risk of transmission. Interrogating the data using the filtering functionality allows simple reports to be produced, such as rapid epidemiological updates to multi-agency and multi-jurisdictional stakeholders. Users are able to tailor this information to inform their surveillance and data requirements, which can be amended and updated to keep up with the rapid changes.

There is growing evidence that a tool such as CRISPER is useful. In some Australian jurisdictions, outbreak investigation and contact tracing processes are de-centralized (39). In these settings, mapping cases at a local level may be useful to assess contact tracing alert locations. A recent mapping application was shown to be useful for those working within the health department in Victoria, however these tools are only available to those working within public health departments (40). Further, it is specific to Victoria and does not provide information for other states and territories, including border towns where contacts are likely to travel.

Additionally, public health alerts from state governments may utilize text messaging or emails to communicate new contact tracing alert locations, outbreaks and changes in restrictions (41). The automated alert system in CRISPER provides detailed summaries by email and future development could include building text messaging functions. Engaging the public with and promoting this functionality could further enhance the usefulness of the tool.

### Limitations

A limitation of CRISPER is that it is only as timely and accurate as the available data sources. While the data engine routinely updates information from these sources, it is possible that some information may be redacted, removed (such as in the case of a false positive or historical infection), and there is little way for the dashboard to explain these changes, or what changes have occurred. This is however an overall limitation of all real-time data, in that it does not look back, and is limited only to the data as-is, accurate at the time it was scraped from the data sources. The system currently relies on publicly available data regarding cases, deaths, tests and contact tracing locations. However, more detailed data have been requested from a number of official sources which will be incorporated into the system if approved. CRISPER is able to incorporate other types of data relevant to COVID-19, e.g., vaccine coverage.

## Conclusion

In summary, we have developed a near real-time interactive dashboard and mapping tool for COVID-19 in Australia using multiple publicly available data sources. We believe integrating data from multiple sources into one platform streamlines access to information and assists public health responders and frontline primary health care providers, as well as optimizes information sharing with the public. We are continuing to work with Australian Government Department of Health to request access to national line-listed data. An evaluation of CRISPER is planned to determine the acceptability of the platform and its usefulness for public health responders and primary health care practitioners. CRISPER provides the foundations for a privacy preserving data sharing platform for other notifiable diseases in Australia which could be used in future public health emergencies.

## Data Availability Statement

The original contributions presented in the study are included in the article/supplementary material, further inquiries can be directed to the corresponding author/s.

## Author Contributions

MH, HW, JS, MP, and GW developed the system. EF, AD, HW, JS, CL, MH, and LM drafted the manuscript. All authors critically reviewed the manuscript and approved the final version. All authors contributed to the design of CRISPER.

## Funding

Funding for this project was provided by a Grant from the Australian Partnership for Preparedness Research on Infectious Disease Emergencies APPRISE (AppID 1116530)—National Health and Medical Research Council (NHMRC) Center for Research Excellence. CLL was supported by an NHMRC Investigator Grant (1193826). LF-K was supported by an NHMRC Fellowship (APP1158469). MS was supported by funding from a Westpac Research Fellowship. GW, MP, and JM were supported by the ANU Software Innovations Institute.

## Conflict of Interest

The authors declare that the research was conducted in the absence of any commercial or financial relationships that could be construed as a potential conflict of interest.

## Publisher's Note

All claims expressed in this article are solely those of the authors and do not necessarily represent those of their affiliated organizations, or those of the publisher, the editors and the reviewers. Any product that may be evaluated in this article, or claim that may be made by its manufacturer, is not guaranteed or endorsed by the publisher.
